# Endogenous Endophthalmitis Secondary to Prostate Abscess

**DOI:** 10.7759/cureus.84765

**Published:** 2025-05-25

**Authors:** Nada A Saleh, Shreeker Patel, Syed Quadri

**Affiliations:** 1 Internal Medicine, Javon Bea Hospital, Rockford, USA; 2 Internal Medicine, University of Illinois, Rockford, USA; 3 Infectious Disease, Javon Bea Hospital, Rockford, USA

**Keywords:** acute endophthalmitis, bacterial endogenous endophthalmitis, immunocompetent patients, intravitreous antibiotics, methicillin-resistant staphylococcus aureus (mrsa)

## Abstract

Endophthalmitis is a severe ophthalmic emergency that necessitates immediate intervention. Most cases are exogenous, originating from eye surgeries, intravitreal injections, or penetrating injuries. Endogenous endophthalmitis occurs when the infection is spread hematogenously, usually as a result of bacteremia. We present a healthy individual with no predisposing factors who presented to the emergency department with decreased left vision.

A male in his late sixties with a medical history of hypertension and hyperlipidemia presented to the ophthalmology clinic with complaints of left eye pain, redness, and decreased vision. Diagnosis of endogenous endophthalmitis was made through fundoscopy, and the patient received intravitreal injections of vancomycin and ceftazidime in the left eye. He was subsequently admitted to the hospital, where blood and urine cultures grew methicillin-resistant Staphylococcus aureus (MRSA). Upon further investigation, the patient reported experiencing obstructive urinary symptoms, prompting the initiation of intravenous (IV) vancomycin and linezolid. CT of the abdomen and pelvis revealed multiple prostatic abscesses. During treatment, the patient's vision gradually improved, and he was discharged with IV antibiotics.

This case emphasizes the importance of prompt diagnosis and treatment in managing this ophthalmologic emergency. A thorough review of systems was essential in identifying the source of bacteremia, enabling timely intervention. We describe the role of magnetic resonance imaging in diagnosing endophthalmitis and highlight the treatment modalities.

## Introduction

Endophthalmitis is an ophthalmic emergency characterized by inflammation of the intraocular cavities and requires immediate diagnosis and intervention to preserve vision [1]. While most cases are exogenous, arising from surgical procedures, intravitreal injections, or ocular trauma, endogenous endophthalmitis results from hematogenous spread of infection, often in the setting of bacteremia or fungemia [1]. This form represents only 2-8% of all endophthalmitis cases, with Staphylococcus aureus and Streptococcus pneumoniae among the most frequently implicated organisms. The first reported case was in 1856, caused by recurrent Klebsiella pneumoniae in a 58-year-old male patient with a history of intravenous drug use (IVDU) [2]. Risk factors for endogenous endophthalmitis include immunosuppression, IVDU, and indwelling catheters [3].

Clinical presentation is variable but may include ocular pain, redness, decreased visual acuity, floaters, and photopsia. Diagnosis often requires a multimodal approach involving clinical examination, fundoscopy, ocular ultrasound, neuroimaging, and culture data. Treatment includes systemic antibiotics as well as supplemental use of intravitreal antimicrobials and, in some cases, vitrectomy [4]. Here, we present a rare case of endogenous methicillin-resistant Staphylococcus aureus (MRSA) endophthalmitis secondary to a prostatic abscess in an immunocompetent patient with no history of IVDU, highlighting the need for a high index of suspicion even in low-risk populations.

## Case presentation

A male in his late sixties with a history of hypertension and hyperlipidemia presented to the ophthalmology clinic with 2-3 days of left eye pain, redness, and decreased vision. He was referred to the emergency department for further evaluation.

Initial symptoms

Two weeks prior to the ocular complaints, the patient reported progressive dysuria, urinary hesitancy, and incomplete bladder emptying. 

Clinical examination

**Figure 1:**
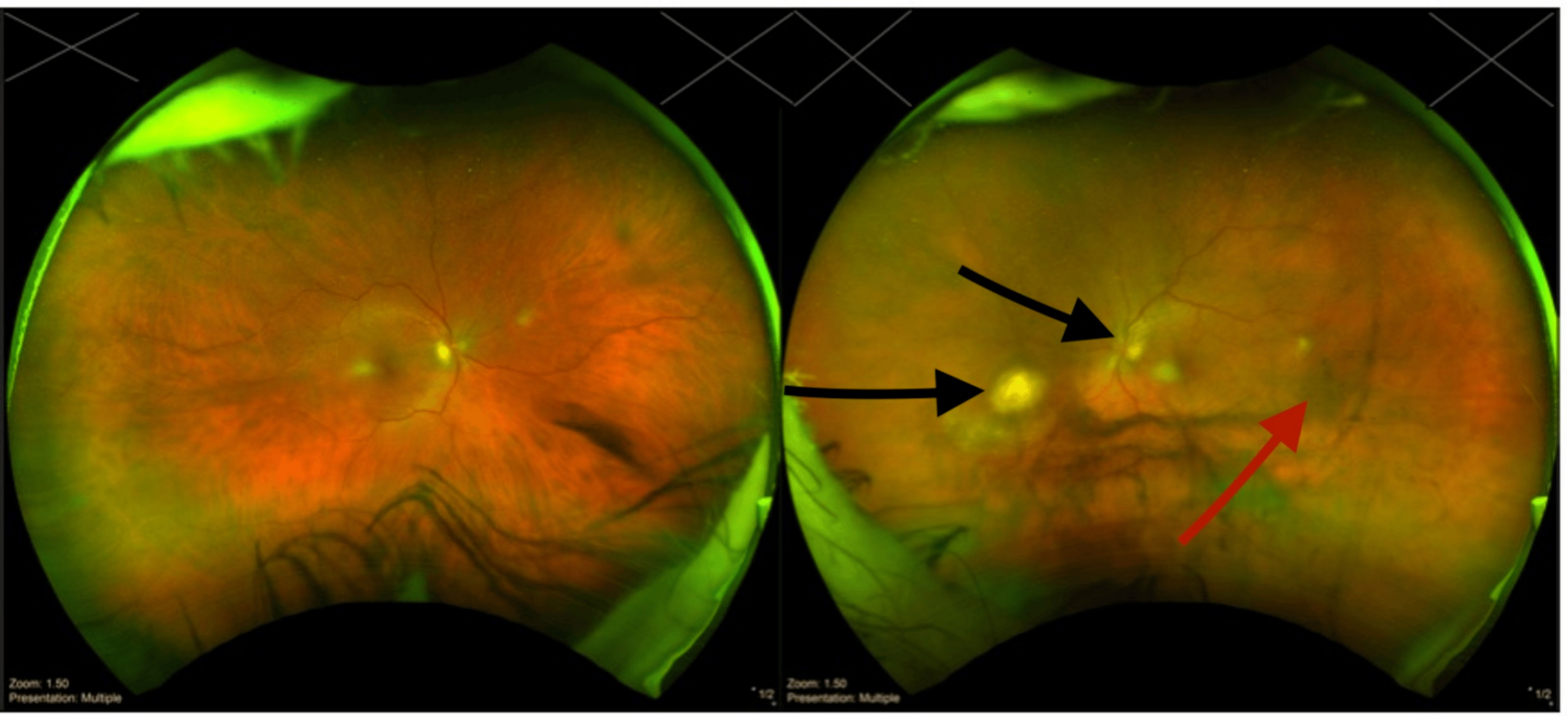
Wide fundus photo of both eyes. Fundus photo of the left eye showing vitreous opacities and vitreous veils (red arrow), which indicates an area of active infection (black arrows)

On arrival, the patient was afebrile and hemodynamically stable. Visual acuity was 20/50 in the right eye and limited to hand motion in the left eye. Intraocular pressure (IOP) was 19 mmHg in the left eye and 15 mmHg in the right. Slit-lamp exam of the left eye revealed a hypopyon, conjunctival injection, and significant vitritis. The fundus exam was notable for left eye vitreous opacities and a choroidal lesion (Figure 1). The retina was poorly visualized with an absent red reflex.

Immediate interventions

The patient received intravitreal vancomycin and ceftazidime, and vitreous fluid was collected for culture. Systemic workup was initiated with blood and urine cultures.

Imaging

**Figure 2:**
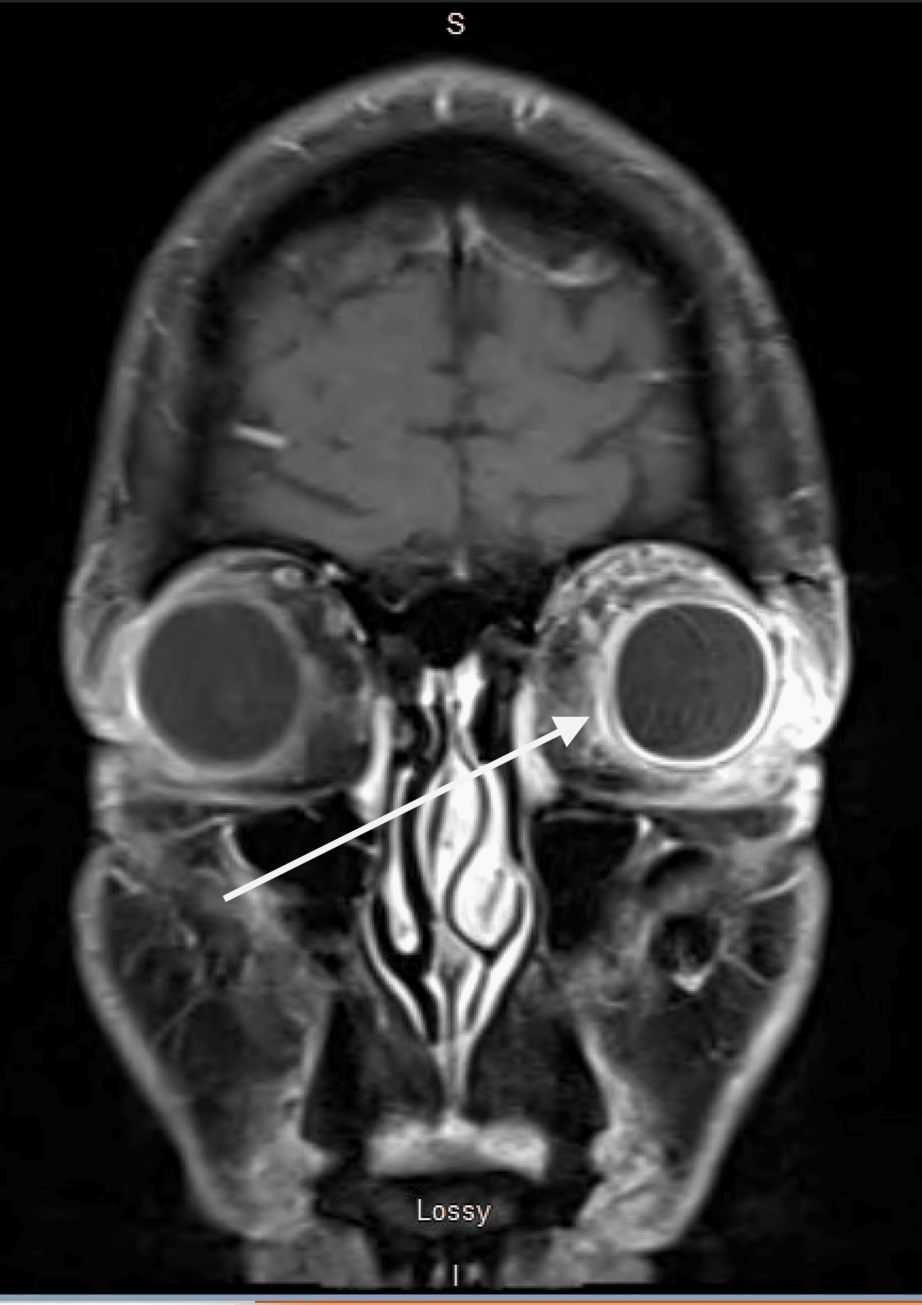
Mild left proptosis, circumferential scleral thickening and enhancement with surrounding orbital fat and edema consistent with endophthalmitis (white arrow)

**Figure 3:**
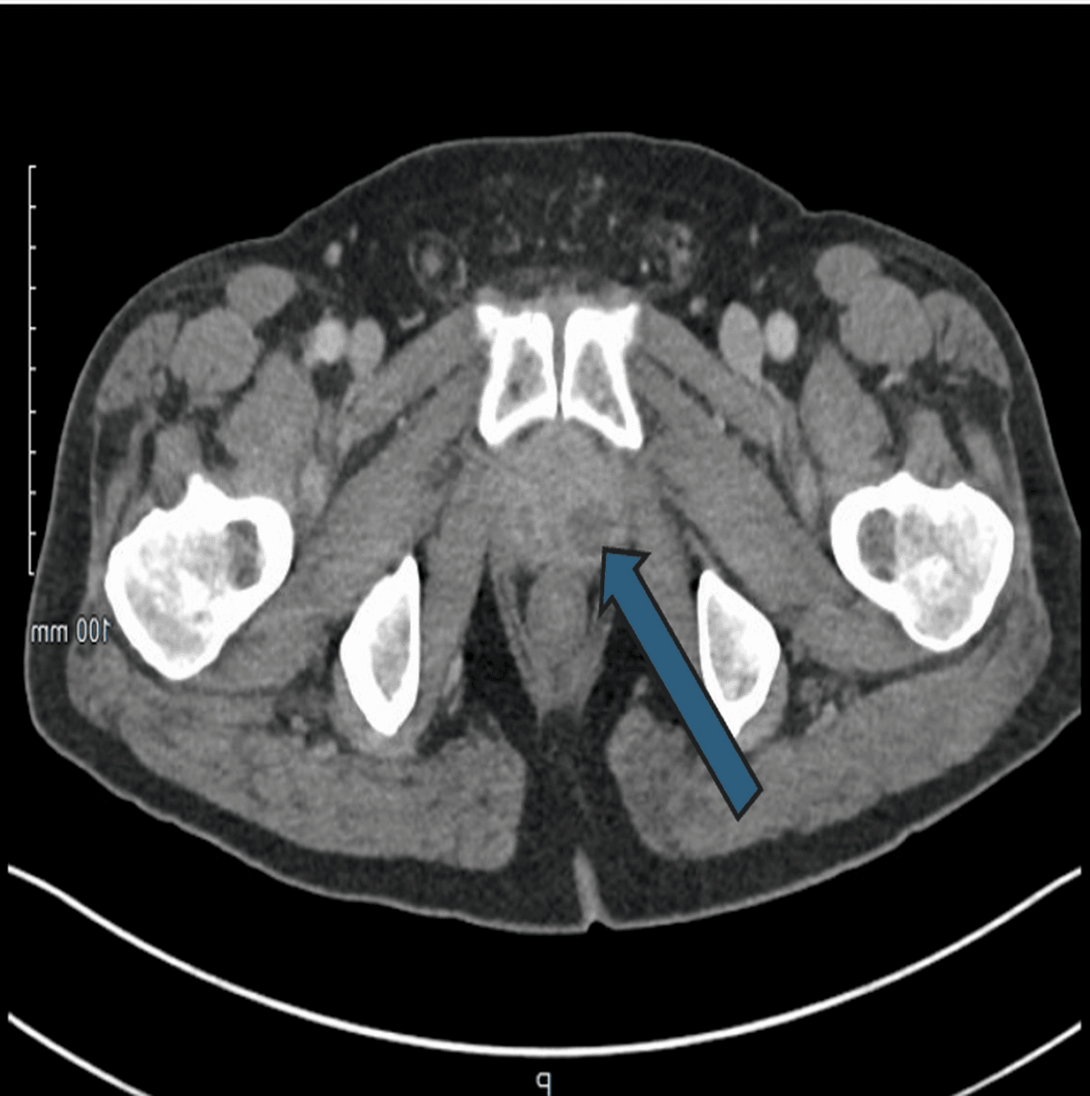
Enlarged prostate with surrounding inflammatory stranding and internal cystic lesions, the largest on the right measuring up to 1.8 cm (marked with the blue arrow)

Brain MRI with and without contrast was pursued to rule out any other sources of infection or intracranial complications. Imaging demonstrated scleral thickening and periorbital edema consistent with endophthalmitis (Figure 2). Abdominopelvic computed tomography revealed multiple prostatic abscesses (Figure 3).

Microbiological confirmation

Both blood and vitreous cultures grew MRSA. Urinalysis showed leukocytes and nitrates; urine culture also confirmed MRSA. Fungal cultures were negative.

Hospital course & outcome

The patient was started on intravenous (IV) vancomycin and linezolid based on susceptibility testing (vancomycin MIC 1, linezolid MIC 2). Echocardiography ruled out infective endocarditis. Serial blood cultures confirmed clearance by day 3. Visual symptoms progressively improved; on day 5, the hypopyon resolved and IOP normalized to 14 mmHg in the left eye. At discharge, the patient had near-baseline visual function. He was discharged on IV vancomycin 1.25 g BID and oral linezolid 600 mg twice a day for a six-week course, with scheduled urology follow-up.

## Discussion

Endogenous endophthalmitis is a rare but vision-threatening infection, typically seen in patients with identifiable risk factors such as IVDU, diabetes, and indwelling catheters [1]. This case is notable due to the absence of traditional risk factors in an otherwise healthy patient. 

Obstructive urinary symptoms likely led to prostatic abscess formation and subsequent hematogenous dissemination of MRSA. The left-sided ocular involvement is atypical. In hematogenous spread, the posterior segment is typically involved, and the right eye is more frequently affected due to direct flow from the right carotid artery [1].

The literature indicates that approximately 40% of endogenous endophthalmitis cases in the USA are due to bacteremia secondary to endocarditis [1]. IVDU is another well-documented cause [5]. A case report from 2022 described a 34-year-old man with a history of IVDU who developed endogenous endophthalmitis secondary to MRSA infection following COVID-19 pneumonia [6].

Most patients with endogenous endophthalmitis present with eye pain and/or decreased vision. However, studies suggest that many patients do not exhibit systemic symptoms (such as fever) or other pertinent physical findings, leading to misdiagnosis. Thus, clinicians should maintain a high index of suspicion when evaluating patients with risk factors such as bacteremia or IVDU who present with ocular complaints. 

Diagnosis can be confirmed through an ultrasound evaluation of the posterior segment, which can help detect vitreous involvement by identifying echoes within the vitreous. Ocular sampling, including vitreous or anterior chamber tap or biopsy, can be performed for culture and microorganism isolation [7]. While fundoscopy and ocular ultrasound remain the mainstays of diagnosis, MRI was employed in this case to rule out intracranial pathology and confirmed orbital involvement. Though not routinely used for diagnosing endophthalmitis, MRI can offer value in complex presentations or in facilities without ophthalmology coverage. MRI may be valuable in ruling out exogenous causes, identifying structural abnormalities, and aiding in cases with an unclear source of infection.

Our patient was treated with a combination of intravitreal and systemic antimicrobials. The most effective agents for achieving therapeutic levels in the vitreous fluid include meropenem, linezolid, and moxifloxacin [8]. Systemic moxifloxacin is a viable option due to its excellent intraocular penetration and broad-spectrum coverage. However, it does not cover MRSA, which makes it unsuitable for this case [9]. Intravitreal antibiotics remain the preferred route, particularly in cases of penetrating injuries [9]. The rationale for combining vancomycin and linezolid, as previously discussed, is their ability to penetrate ocular tissues and their effectiveness against MRSA. However, there is limited literature supporting this specific combination, and further research is needed. Systemic linezolid, which has excellent intraocular penetration, was chosen alongside vancomycin due to the MRSA susceptibility profile. Given the risk of linezolid-associated optic neuropathy, baseline and follow-up visual assessments were performed. The patient remained asymptomatic, and no toxicity was observed during the six-week course.

This case reinforces the importance of early recognition and aggressive treatment, even in atypical presentations. It also illustrates the importance of a comprehensive diagnostic approach-integrating clinical exam, imaging, and culture results.

## Conclusions

Endogenous endophthalmitis is a vision-threatening emergency that requires rapid recognition and aggressive management to prevent irreversible ocular damage and vision loss. This case highlights the importance of maintaining a high index of suspicion for endogenous endophthalmitis in patients with bacteremia and ocular symptoms, even in the absence of classic systemic signs. A delayed or missed diagnosis can lead to devastating complications, including blindness or even loss of the eye. Early clinical suspicion, prompt diagnostic evaluation, and timely initiation of appropriate antimicrobial therapy and surgical intervention, when necessary, are critical in optimizing patient outcomes. Increased awareness among clinicians, especially those managing patients with risk factors such as immunosuppression or IVDU, is essential to improving early detection and treatment. 
